# A simple, rapid, and transgene-free strategy for the generation of transgenic pigs via precise editing of monoclonal porcine fetal fibroblasts: A simple, rapid, and transgene-free strategy for the generation of transgenic pigs

**DOI:** 10.3724/abbs.2025044

**Published:** 2025-05-29

**Authors:** Kun Liu, Nan Huang, Chuanxiang Ding, Qiaoli Lang, Hongyu Chen, Hao Liang, Rendong Fang, Liangpeng Ge, Xi Yang

**Affiliations:** 1 Chongqing Academy of Animal Sciences Chongqing 404100 China; 2 Joint International Research Laboratory of Animal Health and Animal Food Safety College of Veterinary Medicine Southwest University Chongqing 400715 China

Pigs, as crucial economic livestock species, possess remarkable reproductive traits and thus play a highly significant role in promoting the progress of the livestock industry. With the advent and application of CRISPR/Cas9 technology, researchers have explored genetic editing techniques to increase swine reproductive performance, flavour profiles, and nutritional attributes. Additionally, with respect to anatomy, physiology, immunology, and genomics as well as other traits, pigs exhibit remarkable similarities to humans. Genetically edited pigs play crucial roles in human disease models, xenotransplantation, breed improvement, vaccine development, and drug assessment.

Common methods deployed in the preparation of genetically edited pigs include somatic cell nuclear transfer (SCNT), microinjection and sperm-mediated approaches. For example, Shen *et al*. [1] successfully generated *P53*-knockout Diannan miniature pigs using transcription activator-like effector nucleases combined with SCNT, offering a valuable resource for preclinical oncology research. In 2019, Chen *et al*. [2] employed microinjection to deliver Cas9 messenger ribonucleic acid (mRNA) and single guide ribonucleic acid (sgRNA) into the cytoplasm of fertilized eggs. These authors successfully obtained both the albinism phenotype and the combined phenotype of albinism and immunodeficiency in Tibetan miniature pigs. More recently, Tenihara *et al*. [3] introduced the CRISPR/Cas9 protein into fertilized porcine eggs via electroporation, enabling a simple, micromanipulation-free approach for generating gene-edited pigs. Among these methods, SCNT has gained extensive interest among researchers because of its reliability. An essential aspect of SCNT is the preparation of embryonic fibroblasts to serve as donor cells.

Previously, the CRISPR/Cas9 plasmid editing system served as the predominant technique to generate genetically edited embryonic fibroblasts ( Figure 1A) [4]. This approach, which is distinguished by its relative simplicity, high stability, and low cost, was formerly widely utilized in the production of gene-edited pigs. However, plasmid editing is associated with several notable limitations. First, it introduces resistance genes, posing risks of inaccurate gene editing, drug resistance and biosafety concerns. Second, during the CRISPR/Cas9 editing process, there is a possibility of ongoing editing due to deoxyribonucleic acid (DNA) integration. This continuous editing can increase the likelihood of off-target effects, random mutations, and interference with DNA repair mechanisms. Third, the acquisition of positive cell lines via the plasmid editing system typically demands an extended period of *in vitro* cultivation (lasting 3–4 weeks), which increases the risk of apoptosis and chromosomal aberrations. Consequently, plasmid-based transfection is now largely supplanted by ribonucleoprotein (RNP) systems for gene editing. RNP systems bypass plasmids, delivering the Cas9 protein and sgRNA directly into cells, reducing off-target effects and cytotoxicity [5].

**Figure 1:**
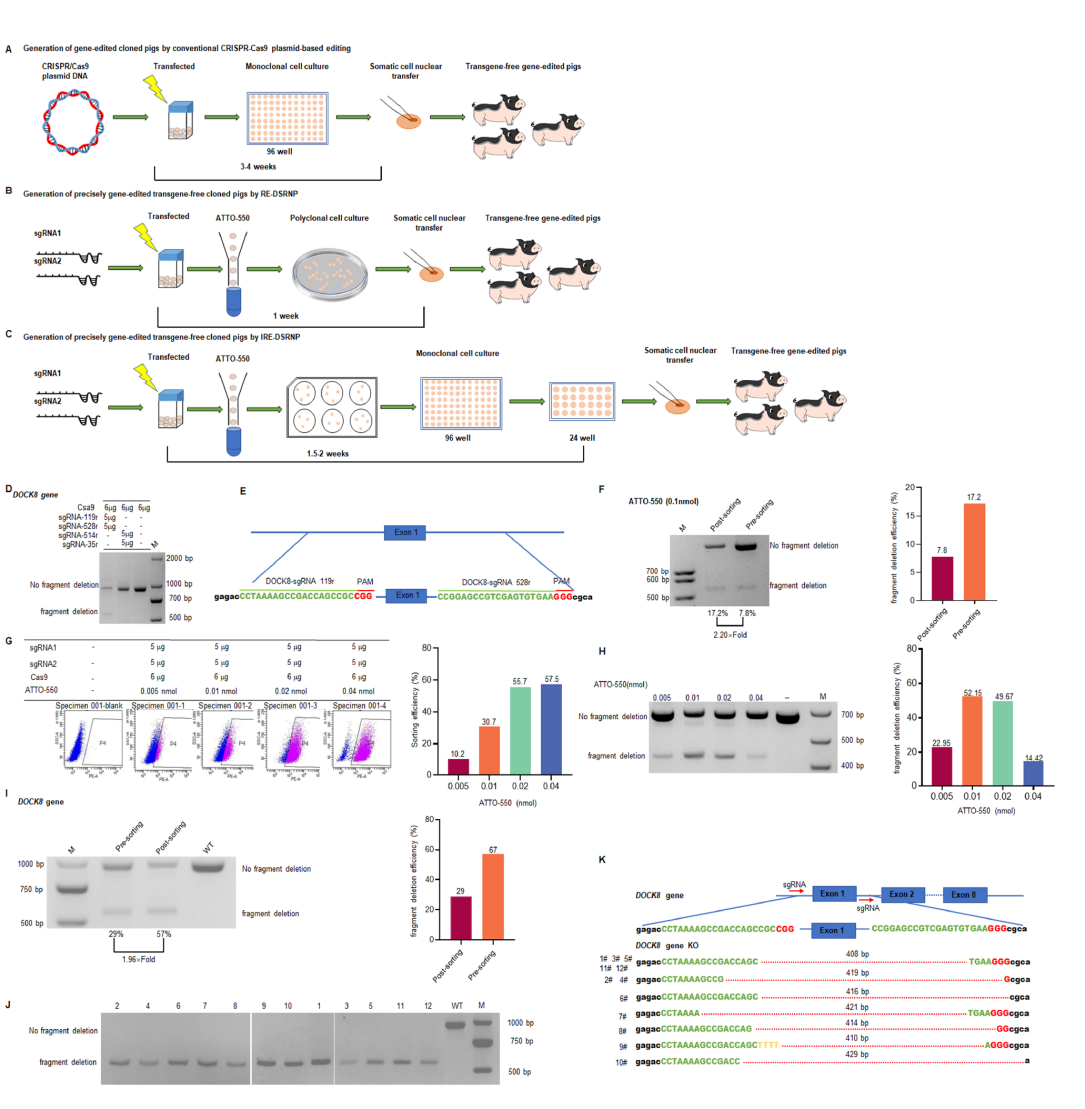
Figure 1 IRE-DSRNP: a more widely utilized method for precisely preparing edited porcine fetal fibroblasts (A) Plasmid editing system: this method involves culturing and genotyping monoclonal cells, a process that typically takes 3–4 weeks. After this period, the appropriate monoclonal cells are selected as donor cells for somatic cell nuclear transfer (SCNT). (B) RE-DSRNP: this method employs dual sgRNAs combined with reporter RNA-enriched RNPs, eliminating the need for monoclonal cell selection and thereby reducing the donor cell generation time from 3–4 weeks to 1 week. The resulting polyclonal cells are then used as donor cells for SCNT. (C) The IRE-DSRNP method, which is an improved RE-DSRNP method. This approach involves monoclonal cell screening in 6-well plates without the use of antibiotic pressure. IRE-DSRNP promotes RE-DSRNP to generate monoclonal cells from polyclonal cells, and the generation time is only prolonged from 1 week to 1.5–2 weeks. (D) Screening for DOCK8 sgRNA. (E) Schematic representation of the two sgRNA target sites in exon 1 of the pig DOCK8 gene. The exon 1 region is shown in blue, the protospacer sequences are highlighted in green, and the PAM sequences are indicated in red. (F) Assessment of editing efficiency by PCR before and after flow cytometry sorting. (G) Optimization of ATTO-550 concentration. (H) PCR-based identification of editing efficiency in enriched cells across different ATTO-550 concentration groups. (I) PCR analysis of editing efficiency before and after enrichment by fluorescence-activated cell sorting (FACS) using the optimal concentration of ATTO-550. (J) PCR identification of positive cells following enrichment. (K) Sanger sequencing of DOCK8-knockout cell lines. The exon regions are indicated by blue rectangles, the protospacer sequences are shown in green, the PAM sequences are depicted in red, and the mutant bases are highlighted in yellow.

In 2022, Xu *et al*. [6] developed the reporter RNA-enriched dual-sgRNA CRISPR/Cas9 ribonucleoprotein (RE-DSRNP) method, a transgene-free approach using CRISPR/Cas9 RNPs enriched with ATTO ^TM^550-tracrRNA (IDT, Iowa, USA) as a fluorescent RNA probe ( Figure 1B). This method reduced the time needed to generate donor cells from 3-4 weeks to one week, resulting in high-efficiency *WIP1* gene knockouts and the production of pigs with male reproductive disorders. However, owing to genetic diversity, not all target genes achieve 95% editing efficiency, as demonstrated by the RE-DSRNP method, with some falling below 90%. For example, DOCK8, which belongs to the DOCK family, is an atypical guanine nucleotide exchange factor that plays a crucial role in immune responses. DOCK8 deficiency syndrome, a rare hereditary disorder, often leads to combined immunodeficiency and is characterized by elevated serum immunoglobulin E levels, increased eosinophil counts, and immunodeficiency-related symptoms [7]. To better understand the pathogenic mechanism of DOCK8 deficiency, disease models capable of accurately elucidating its pathogenesis are urgently needed. In this study, we targeted exon 1 of the *DOCK8* gene to construct a knockout pig model. Four gRNAs were designed on the basis of the *DOCK8* gene sequence and paired into two sets of sgRNAs to assess gene editing efficiency ( Supplementary Table S1). The primers used for PCR amplification are shown in Supplementary Table S2. The results demonstrated that only the pairing of *DOCK8*-gRNA 119r and *DOCK8*-gRNA 528r successfully targeted the gene ( Figure 1D). Specifically, “ *DOCK8*-gRNA 119r” is located at the 5′ untranslated region of the exon, whereas “ *DOCK8*-gRNA 528r” resides in the immediate downstream intron ( Figure 1E). Gene editing efficiency tests performed on primary porcine fetal fibroblasts (PFFs) from Bama pigs revealed an initial editing efficiency of 7.8%.

To increase editing efficiency, we adopted the RE-DSRNP method and adjusted the concentration of ATTO-550 to 0.1 nmol for cell sorting enrichment. This method achieved a 2.2-fold increase in editing efficiency, reaching 17.2% ( Figure 1F). However, this efficiency remains insufficient for direct use in SCNT. We further optimized the concentration of ATTO-550 to values of 0.005, 0.01, 0.02, and 0.04 nmol to maximize efficiency. As shown in Figure 1G, increasing the ATTO-550 concentration resulted in a continuous increase in the proportion of positive cells, from 10.2% in the 0.005 nmol group to 57.5% in the 0.04 nmol group. Genomic DNA was extracted from enriched positive cells, and the gene targeting efficiency was assessed using PCR. As shown in Figure 1H, the editing efficiency initially increased but then decreased with increasing ATTO-550 concentration. At 0.005 nmol, the editing efficiency was 22.95%; at 0.01 nmol, it peaked at 52.51%. The efficiency gradually decreased to 49.67% at 0.02 nmol and decreased further to 14.42% at 0.04 nmol. Our results revealed a nonlinear correlation between ATTO-550 concentration and editing efficiency. On the basis of these results, we determined that an ATTO-550 concentration of 0.01 nmol achieved the highest editing efficiency (52.51%).

However, polyclonal cells with an editing efficiency of 52.51% were unsuitable for direct application in SCNT. Additionally, further optimization of the ATTO-550 concentration failed to achieve the required editing efficiency of 95% or higher. This limitation poses a significant challenge for downstream SCNT procedures. To address these challenges, we developed an improved method, improved reporter RNA-enriched dual-sgRNA CRISPR/Cas9 ribonucleoproteins (IRE-DSRNPs), which integrates the strengths of existing approaches while addressing their limitations. IRE-DSRNP involves co-incubating the Cas9 protein with dual sgRNAs, introducing ATTO-550 and electroporation into PFFs, followed by fluorescence-activated cell sorting 24 h after electroporation and the culture of monoclonal cells in 6-well plates instead of traditional 96-well plates to increase growth and quality ( Figure 1C). The IRE-DSRNP method eliminates the introduction of resistance genes and avoids antibiotic selection, thereby minimizing external interference with cell growth. This approach significantly reduces the time required to establish monoclonal cell lines from 3–4 weeks to approximately 1.5–2 weeks, accelerating the production of gene-edited donor cells. Although the establishment of cell lines takes slightly longer than the RE-DSRNP method does, the IRE-DSRNP protocol facilitates the production of high-quality monoclonal edited cell lines with enhanced editing efficiency for SCNT applications ( Table 1).

**Table:** **Table 1** Comparison among IRE-DSRNP, RE-DSRNP and conventional plasmid-based editing for transgenic pig generation

	Conventional plasmid-based editing	RE-DSRNP	IRE-DSRNP
Transgene-free	No	Yes	Yes
Increases editing efficiency by enrichment	No	Yes	Yes
Generation time of donor cells	3–4 weeks	1 week	1.5–2 week
Off-target effect screening	Detectable effects	None detected	Detectable effects
Monoclonal condition	Monoclonal	Polyclonal	Monoclonal

To obtain donor cells with a nearly 100% positive rate, we employed the IRE-DSRNP methodology for the preparation of *DOCK8* donor cells. In detail, in the process of generating *DOCK8*-KO ( *DOCK8* knockout) donor cells, 0.01 nmol ATTO-550 was co-transferred along with Cas9 (NEB, Ipswich, USA) RNPs into PFFs. This transfer was carried out using a Nucleofector 2b device (Lonza, Cologne, Germany) and a Basic Nucleofector™ kit specifically designed for primary mammalian fibroblasts (Lonza). Following an incubation period of 24 h, the PFFs were treated with 0.05% trypsin-EDTA solution to facilitate cell digestion, which was essential for subsequent cell sorting procedures. The top 15% of positively stained cells were enriched during sorting. As shown in Figure 1I, the presorting editing efficiency was approximately 29%, which increased to 57% postsorting, representing a 1.96-fold increase. The enriched cells were subjected to subcloning in 6-well plates, with 500 cells seeded per well. The culture medium was replaced by fresh medium on the fourth day. The monoclonal cells were subsequently selected by cloning rings. They were initially cultured and expanded in 96-well plates and then transferred to 24-well plates for further expansion and continuous cultivation. Several *DOCK8*-KO cell lines were successfully generated, and partial monoclonal PCR results are shown in Figure 1J. To validate the gene editing, twelve monoclonal cell lines were selected, and PCR amplicons were analyzed by Sanger sequencing. Alignment to the reference genome confirmed the successful deletion of *DOCK8* exon 1 ( Figure 1K).

To provide additional evidence of the efficacy of the IRE-DSRNP method, we utilized this approach to create *IgA*-knockout pigs. IgA, a key component of mucosal immunity, is critical for preventing severe immunodeficiency diseases, including infections, autoimmune diseases, and intestinal inflammatory diseases [8]. We aimed to knock out three domains of the *IgA* gene (CH1, CH2, and CH3) and designed six sgRNAs targeting CH1 and CH3 ( Figure 2A). Pairwise targeting tests were performed, and the gene targeting efficiencies of the 6 sgRNA combinations were evaluated ( Supplementary Table S3). The most efficient sgRNA pair (sgRNA-486r/sgRNA-1514r) was selected for subsequent experiments ( Figure 2B). Multiple positive knockout cells were successfully generated using the IRE-DSRNP method ( Figure 2C). After sequencing, three distinct types of gene-deficient cells were identified, with deletions of 1044 bp, 1043 bp, and 1039 bp in the CH1-CH3 regions, respectively ( Figure 2D). Notably, the cells with a 1044 bp deletion are heterozygous, whereas the others are homozygous.

**Figure 2:**
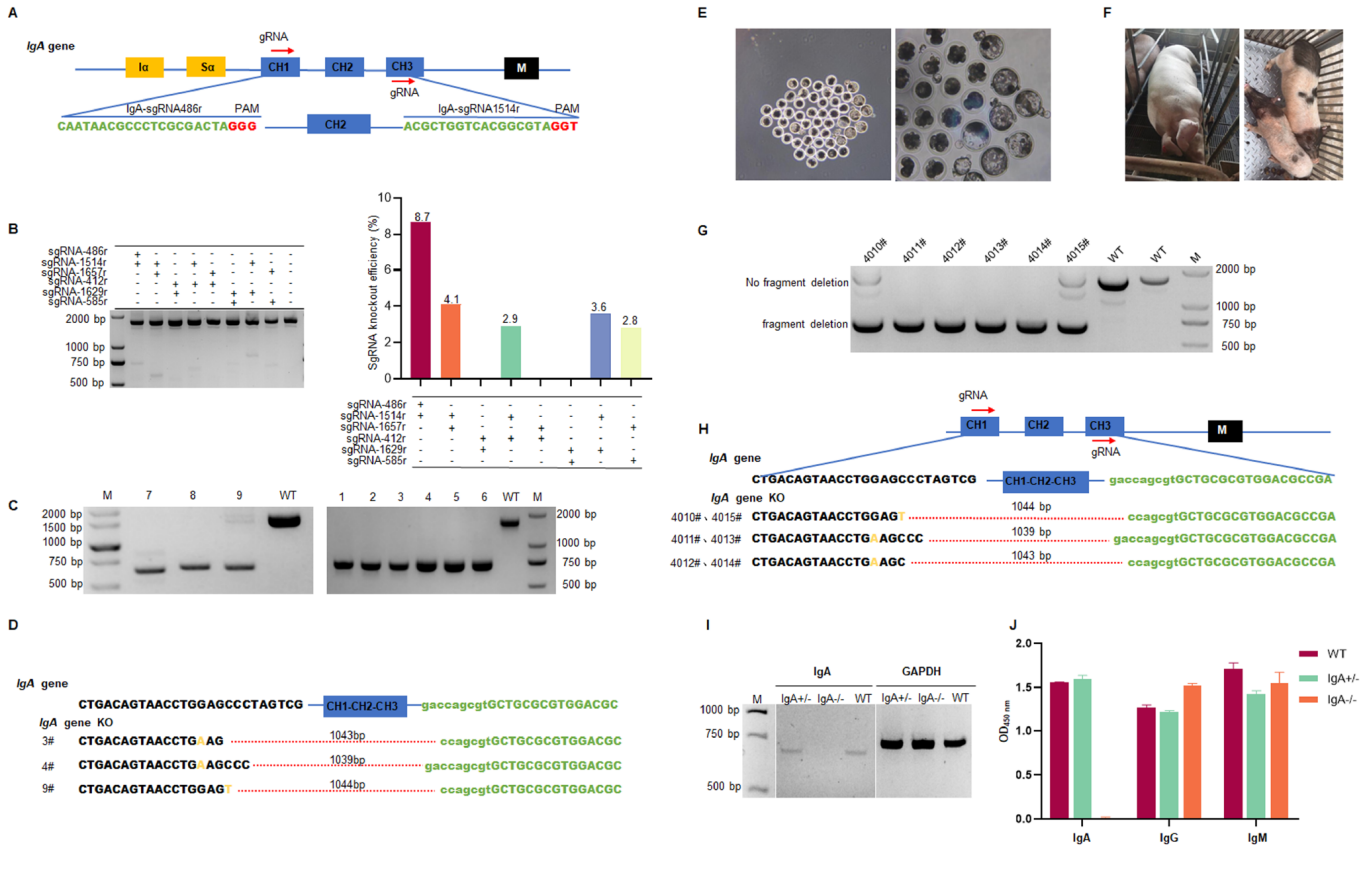
Figure 2 Rapid and effective generation of *IgA*-knockout pigs via the IRE-DSRNP method (A) Knockout strategy for the IgA gene. Schematic diagram showing two sgRNA target sites in the CH1 and CH3 regions of the porcine IgA gene. The CH1, CH2, and CH3 regions are represented by blue rectangles; the Iα and Sα regions are represented by yellow rectangles; the M region is represented by a black rectangle; the protospacer sequence is represented by green; and the PAM sequence is represented by red. (B) PCR analysis of knockout efficiency using paired sgRNA (5 μg each) and 6 μg of Cas9. (C) PCR-based identification of IgA-positive cell lines (representative data). (D) Sanger sequencing of IgA-knockout cell lines, revealing three distinct types of deletions in the cell lines. (E) Schematic diagram illustrating the in vitro development of gene-edited cloned embryos. (F) Photograph of surrogate sows after embryo transfer surgery and F0 generation IgA-deficient piglets. (G) PCR-based identification of six F0 generation piglets. (H) Sanger sequencing for genotype identification of F0 generation piglets. (I) RT-PCR analysis of IgA mRNA expression in the spleens of F0 generation piglets [4010# (IgA–/+) and 4012# (IgA–/–)]. (J) ELISA measurement of serum IgA, IgG, and IgM levels in IgA-deficient piglets; the results are from three independent experiments.

To further confirm the effectiveness of this approach, we performed SCNT to generate *IgA* gene knockout pigs. To obtain F0-generation piglets with multiple genotypes simultaneously, we intentionally mixed the three genotypes of cells to serve as donor cells. Three positive cells were individually injected into the perivitelline cytoplasm of enucleated oocytes to form reconstructed embryos. The three types of reconstructed embryos were subsequently transferred together into the oviduct of a surrogate female through surgical methods, with the aim of producing piglets with multiple genotypes. A total of 880 fresh oocytes were harvested from the slaughterhouse, among which 660 mature oocytes were carefully selected. Nucleus removal and donor cell nuclear transfer were performed on 500 oocytes, resulting in 400 fused cells after activation. Using embryo transfer technology, 300 embryos were transplanted into a surrogate sow ( Supplementary Table S4). Figure 2E shows the *in vitro* development of *IgA* gene knockout cloned embryos. After the procedure, the surrogate sow was transferred to a designated enclosure for rearing. A B-ultrasound examination conducted 28 days posttransplantation revealed that surrogate sow 928# successfully became pregnant. After 143 days of gestation, the sows gave birth to six piglets numbered 4010#, 4011#, 4012#, 4013#, 4014#, and 4015#. Figure 2F shows the surrogate sows housed in farrowing crates following transplantation surgery and the F0 piglets born. DNA extracted from ear tissues of the piglets was subjected to PCR amplification for genotyping. The results indicated that piglets 4010# and 4015# were heterozygotes, whereas the remaining piglets were homozygous knockouts ( Figure 2G). Sequencing and comparison of the *IgA* gene sequence revealed large fragment deletions in the CH1-CH3 region, which was consistent with expectations. Fortunately, the deliberate transplantation of cells with different genotypes enabled us to successfully obtain F0-generation piglets of three distinct genotypes, with deletions of 1044, 1043, and 1039 base pairs ( Figure 2H). Additionally, two piglets of each genotype were obtained, resulting in the successful generation of the IgA-deficient Bama pig model.

To assess potential off-target effects caused by unintentional cleavage of the CRISPR/Cas9 system, we analyzed the pig genome and predicted multiple potential off-target sites (OTSs) using the online CRISPOR website ( http://crispor.tefor.net/), and the results are shown in Supplementary Table S5. The ten leading off-target sites were selected for additional examination, and specific PCR primers were consequently developed ( Supplementary Table S6). Specifically, three base differences were found at OTS2 and OTS4, and one base difference was found at OTS8, OTS10, and OTS1. We consider this phenomenon normal, as the database template sequence originated from Duroc pigs, whereas our study used Bama pigs. However, compared with the WT sequencing results, we confirmed that the knockout piglets presented no additional changes at the predicted off-target sites, indicating that no off-target effects occurred ( Supplementary Figure S1–S19).

To assess the expression level of IgA in *IgA*-knockout pigs, we employed RNeasy Mini kit (Qiagen, Hilden, Germany) to extract splenic mRNA from transgenic pigs of two distinct genotypes, namely, IgA ^+/–^ and IgA ^–/–^. PCR amplification revealed that 4010# (IgA ^+/–^) still presented IgA mRNA expression, whereas pig 4012# (IgA ^–/–^) presented barely detectable IgA expression ( Figure 2I). Furthermore, we detected the protein expression levels of IgA, IgG, and IgM in the serum using the ELISA method. The results revealed that IgA protein was undetectable in the serum of pig 4012# with the IgA ^–/–^ genotype ( Figure 2J), whereas the levels of IgG and IgM proteins were within the normal range. In conclusion, we successfully generated *IgA*-knockout pigs using the IRE-DSRNP method.

The IRE-DSRNP method has the potential to be widely applied to most cell types and genetic editing in large animals. This method outperforms traditional plasmid editing methods and is particularly advantageous for primary cells, which often cannot sustain prolonged *in vitro* growth. This is because plasmid DNA is toxic to primary cells [9]. Additionally, both DNA and RNA can trigger innate cellular responses, leading to the premature death of primary cells. In contrast, RNPs do not have these drawbacks, making them especially suitable for editing primary cells. The IRE-DSRNP method can be employed for routine gene editing as well as for genes with low editing efficiencies using conventional methods. We recommend using the IRE-DSRNP method to obtain highly efficient and accurate gene-edited donor cells. This approach is expected to provide robust technical support for SCNT donor cell preparation and to drive advancements in genetic editing technologies.

A limitation of our study is the incomplete understanding of the mechanism of ATTO-550. While our results demonstrate that the addition of ATTO-550 significantly enhances gene editing efficiency, the precise molecular mechanism remains unclear. For example, in *DOCK8* gene editing, the optimal sgRNA achieved an editing efficiency of only 7.8%. However, with the addition of 0.1 nmol ATTO-550, the efficiency increased to 17.2%. To further optimize gene editing, we systematically explored the effects of varying ATTO-550 concentrations. Our results showed that editing efficiency does not correlate directly with the enrichment efficiency of cell sorting. Specifically, editing efficiency initially increased but subsequently decreased when the ATTO-550 concentration exceeded a certain threshold, suggesting that excessive ATTO-550 levels inhibit gene editing. Further investigations are necessary to elucidate the molecular mechanism underlying this effect and to optimize the application of ATTO-550 in gene editing.

In summary, IRE-DSRNP represents an efficient, transgene-free gene editing technique that enhances editing efficiency and reduces preparation time for genetically modified donor cells. This method was successfully applied to produce *DOCK8*- and *IgA*-knockout PFFs. Using SCNT and embryo transfer, we successfully generated six *IgA* gene knockout pigs. This achievement not only attests to the reliability of the IRE-DSRNP method in the production of transgenic pigs but also lays a solid foundation for further in-depth investigations into IgA function and related therapeutic applications.

## Supporting information

25070Supplementary_Data

## Supplementary Data

Supplementary data is available at *Acta Biochimica et Biophysica Sinica* online.
