# Supplementary material for: A simple, rapid, and transgene-free strategy for the generation of transgenic pigs via precise editing of monoclonal porcine fetal fibroblasts: A simple, rapid, and transgene-free strategy for the generation of transgenic pigs
Source: Acta Biochim Biophys Sin (Shanghai). 2025 May 29;57(10):1732–6. doi: 10.3724/abbs.2025044 (PMC12616732; doi:10.3724/abbs.2025044)
Supplement: 25070Supplementary_Data [file 25070Supplementary_Data.docx]

**Supplementary Table S1. sgRNA oligonucleotide chain of *DOCK8* gene**

| Name | Sequence |
| --- | --- |
| *DOCK8*-sgRNA-119r | 5′-CCTAAAAGCCGACCAGCCGCCGG-3′ |
| *DOCK8*-sgRNA-528r | 5′-CCGGAGCCGTCGAGTGTGAAGGG-3′ |
| *DOCK8*-sgRNA-514r | 5′-CCTGCGCCCTTCACACTCGACGG-3′ |
| *DOCK8*-sgRNA-35r | 5′-TCGCTGCAGCGCATGCCGAGCGG-3′ |

**Supplementary Table S2. Sequences of primers for the target fragment**

| Name | Sequences | Amplicons (bp) |
| --- | --- | --- |
| *DOCK8* | F: 5'-GAAGTTTCCAAACGCGACTGACAGA-3'  R: 5'-AAGCACCTCTGGTGAGAACTTTGAG-3' | 1724 |
| *IgA* | F: 5'-CTCTGACAGTGCCCAAAGATTCAGT-3'  R: 5'-CTTCAAGCGAGCACGGAGTTTATTC-3' | 970 |
| *IgA* (RT-PCR) | F: 5'-AGAGACATTCTCCTGCACCG-3'  R: 5'- CGTTGACGTGGGTGGGTTTA-3' | 600 |
| *GAPDH* (RT-PCR) | F: 5'- CAAGGTCATCCATGACAACTTTG -3'  R: 5'- GTCCACCACCCTGTTGCTGTAG -3' | 496 |

**Supplementary Table S3. sgRNA oligonucleotide chain of *IgA* gene**

| Name | Sequence |
| --- | --- |
| *IgA*-sgRNA-486r | 5′-CCTAAAAGCCGACCAGCCGCCGG-3′ |
| *IgA*-sgRNA-1514r | 5′-CCGGAGCCGTCGAGTGTGAAGGG-3′ |
| *IgA*-sgRNA-1657r | 5′-CCTGCGCCCTTCACACTCGACGG-3′ |
| *IgA*-sgRNA-412r | 5′- GCGATGACCACATATCCGGCAGG-3′ |
| *IgA*-sgRNA-1629r | 5′- TTTACCCGCCAGGCGGTCGATGG-3′ |
| *IgA*-sgRNA-585r | 5′- ACTTCAGGATCTGATCGGCTGGG-3′ |

**Supplementary Table S4. Generation of *IgA*-knockout pigs by SCNT**

| Total oocyte count | Number of mature  oocytes | Number of enucleated oocytes | Number of confluent cells | Number of SCNT  transplanted cells |
| --- | --- | --- | --- | --- |
| 880 | 640 | 500 | 400 | 300 |
| 928# days of  pregnancy | Number of births | Homozygous piglets | Heterozygous piglets | Surviving piglets |
| 143 | 6 | 4 | 2 | 6 |

**Supplementary Table S5. Analysis of potential off-target sites**

| Locus | Sequences | Presence of potential off-target |
| --- | --- | --- |
| IgA-gRNA-1514r | 5'-ACGCTGGTCACGGCGTAGGT-3' |  |
| gRNA-OTS1 | 5'-ACGATGGTCACAGCGAAGAT-3' | NO |
| gRNA-OTS2 | 5'-ACGCGGGTCACAGCATAGAT-3' | NO |
| gRNA-OTS3 | 5'-ATGGTGGCCACGGTGTAGGT-3' | NO |
| gRNA-OTS4 | 5'-CCGCAGGTCACGGTGAAGGT-3' | NO |
| gRNA-OTS5 | 5'-AGGCTGGTCTCAGAGTAGGT-3' | NO |
| gRNA-OTS6 | 5'-CTGCTGGTCACGGTGGAGGT-3' | NO |
| gRNA-OTS7 | 5'-GCGGTGGCCATGGCGTAGGT-3' | NO |
| gRNA-OTS8 | 5'-CGGCTGGTCCCGGCGCAGGT-3' | NO |
| gRNA-OTS9 | 5'-ATTCTGGTCACTGTGTAGGT-3' | NO |
| gRNA-OTS10 | 5'-AGGCTGGCCAGGGCGCAGGT-3' | NO |
| IgA-gRNA-486r | 5'-CAATAACGCCCTCGCGACTAGGG-3' |  |
| gRNA-OTS11 | 5'-CCATACGGCCCTCGCGCCTA-3' | NO |
| gRNA-OTS12 | 5'-CAATCAAGCCCTGGAGACTA-3' | NO |
| gRNA-OTS13 | 5'-CAATAACCCCCTCTAGACCA-3' | NO |
| gRNA-OTS14 | 5'-CAATAAACCCCTCTTGACTA-3' | NO |
| gRNA-OTS15 | 5'-AAATAAAGCCCTGGGGACTA-3' | NO |
| gRNA-OTS16 | 5'-CAACAACGCCCTCTCCACTA-3' | NO |
| gRNA-OTS17 | 5'-CATTAACGACCGCGCCACTA-3' | NO |
| gRNA-OTS18 | 5'-CATTAACCACCTCGCCACTA-3' | NO |
| gRNA-OTS19 | 5'-CAGTAACTCCCTCGCCGCTA-3' | NO |

**Supplementary** **Table S6. Primers used for off-target analysis**

| Primers | Sequences (5'→3') | Amplicons (bp) |
| --- | --- | --- |
| gRNA-1514r-OTS1 | F: CCAGTACGAGCGGATCTTCAG  R: GGATAGGATGCCGCATAGCATG | 548 |
| gRNA-1514r-OTS2 | F：CGTGCACCCACGTACACACA  R：AAAGGGAGTCACCTCGTGTTTG | 556 |
| gRNA-1514r-OTS3 | F: GAACCCTAGCTCACTCGTGGT  R: CCACCAGTTTCTCGCGGTTG | 425 |
| gRNA-1514r-OTS4 | F: TGGGGTGTGGTCAAGGTCA  R: CCTTCTTGCACTCGCCGAATAC | 517 |
| gRNA-1514r-OTS5 | F: GCAAGACAGTTCTCCATCAGCA  R: GGTTTTAAAACCGTAAGGCAAGCC | 462 |
| gRNA-1514r-OTS6 | F: GCACCAGCAGACCTCCTTCT  R: GGTAGGGATAACCCCTGCA | 551 |
| gRNA-1514r-OTS7 | F: GTGAGGAGATTATGTAACTTGCGT  R: TCCAGGGCAAGAGTGTCTAATTC | 483 |
| gRNA-1514r-OTS8 | F: CAGCAAGTCGTCCGAGTGTTT  R: GTTTAGGGCAACCCAGGCA | 538 |
| gRNA-1514r-OTS9 | F: GGGCTGGCTACTAATGTTTGC  R: GGTGAGAGGTTGTATAGATGCACG | 555 |
| gRNA-1514r-OTS10 | F: GGCCATTTCCTGTTTTTCTCCCA  R: ATCAACACAGCCGCTCCA | 462 |
| gRNA-486r-OTS11 | F: CCAGGAGAGGTAATTACCCGC  R: GAGGCCAGAGCTCGGAGTTAA | 358 |
| gRNA-486r-OTS12 | F: GTCCTCAAGTCCATGAACTCTTGC  R: TTTCCCCTCAGGAGTTGCTTCC | 675 |
| gRNA-486r-OTS13 | F: GAGCAATAGGGGCTAAGTGC  R: TTCCAACTGGTGAAACAGGGT | 390 |
| gRNA-486r-OTS14 | F: GCCTGAATGACAGAACTGACC  R: TGGAGAGGCAGCGTAGG | 559 |
| gRNA-486r-OTS15 | F: ATCCGAGGGGGGTAGTT  R: AGATGGCACCCAGAAAGATG | 614 |
| gRNA-486r-OTS16 | F: CGGTGGACTTGATTGGATGT  R: CCATGTTCCTGAATGAAGTCACA | 702 |
| gRNA-486r-OTS17 | F: CAATAAACCCTTAGTCACACTGAT  R: AGTGAAACCAACTTGAGCTAGAG | 595 |
| gRNA-486r-OTS18 | F: GGTGGGCAAATGTTACTTCAAGT  R: TTCTTAGACATTGCACTGCTCT | 523 |
| gRNA-486r-OTS19 | F: GCAGATGATCTCCCCAGATGT  R: GTCTGAGCCTGAGCATTAGAG | 647 |


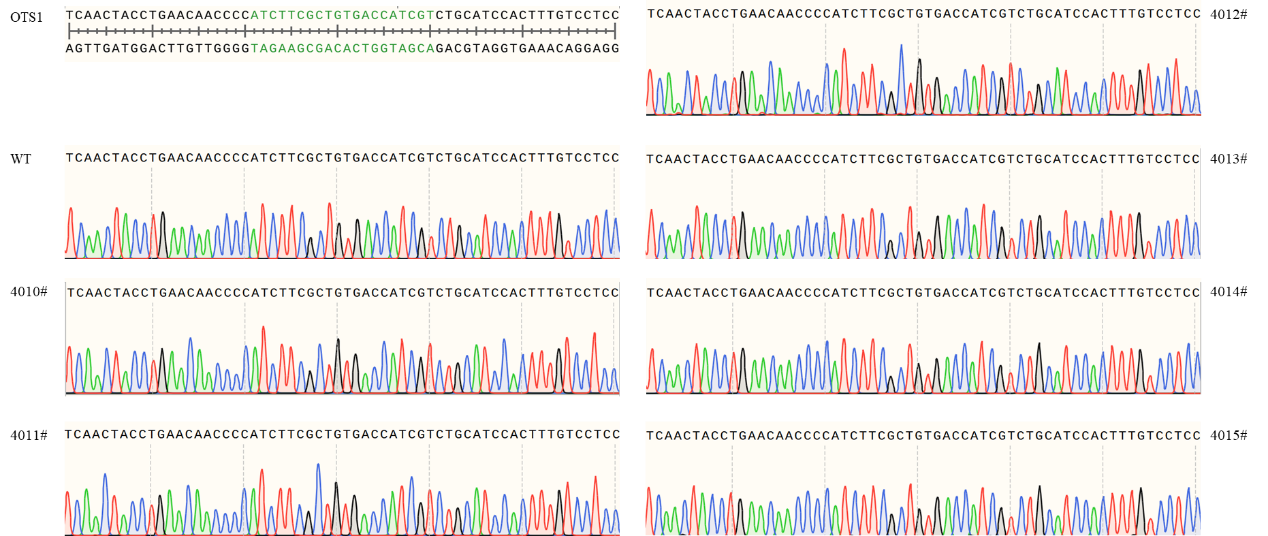


**Supplementary Figure S1. Sequencing results of OTS1 (NC_010461.5:122711877-122711899)**


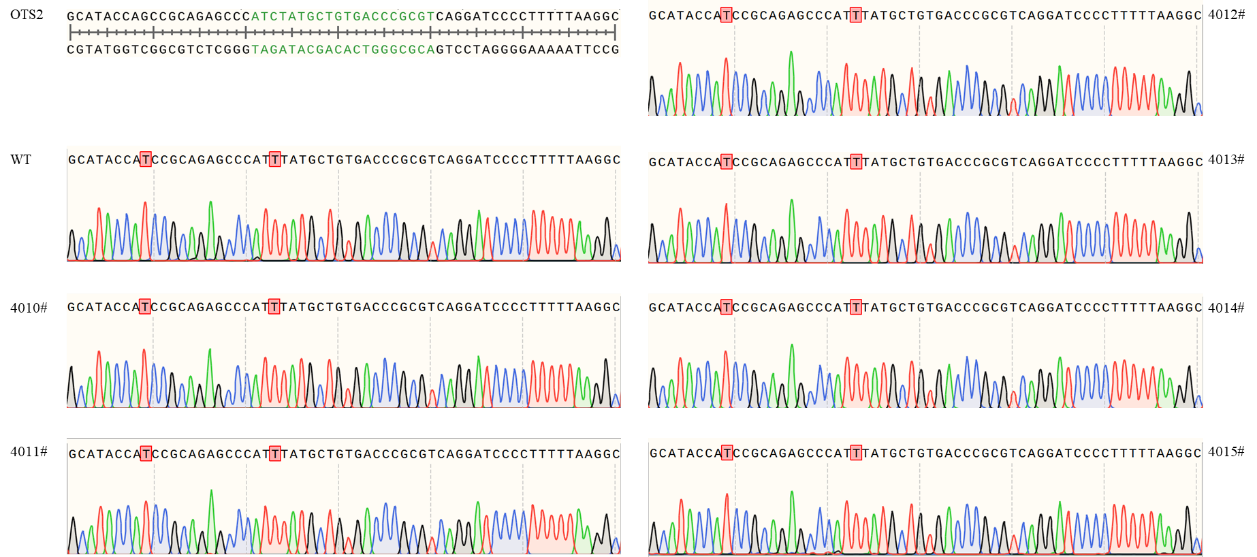


**Supplementary** **Figure S2. Sequencing results of OTS2 (NC_010453.5:77909130-77909152)**


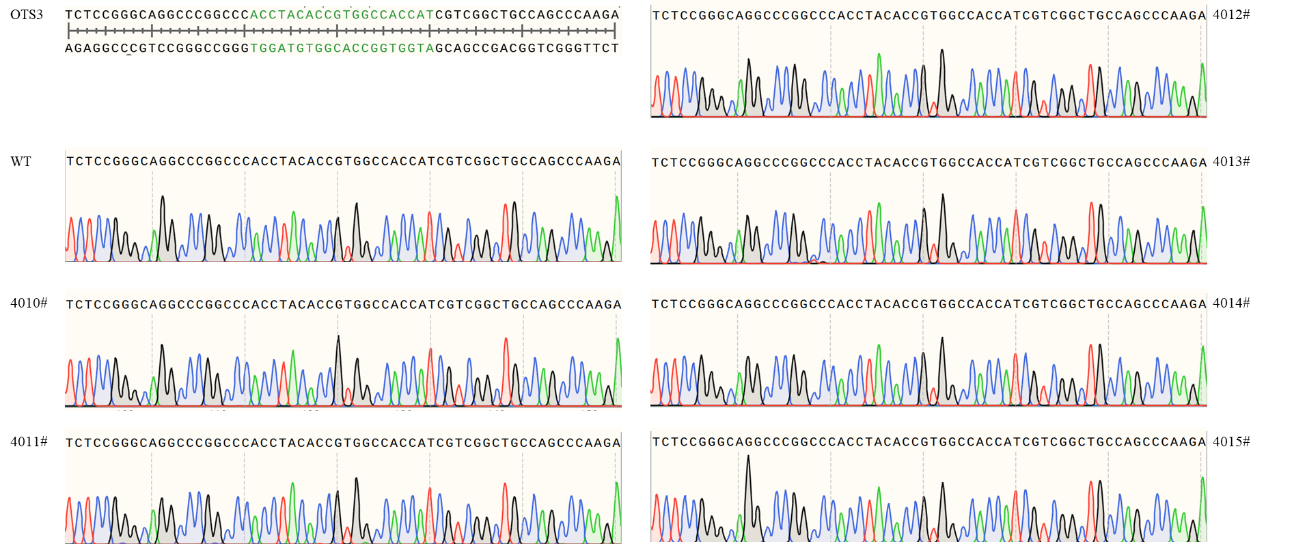


**Supplementary** **Figure S3. Sequencing results of OTS3 (NC_010444.4:70658457-70658479)**


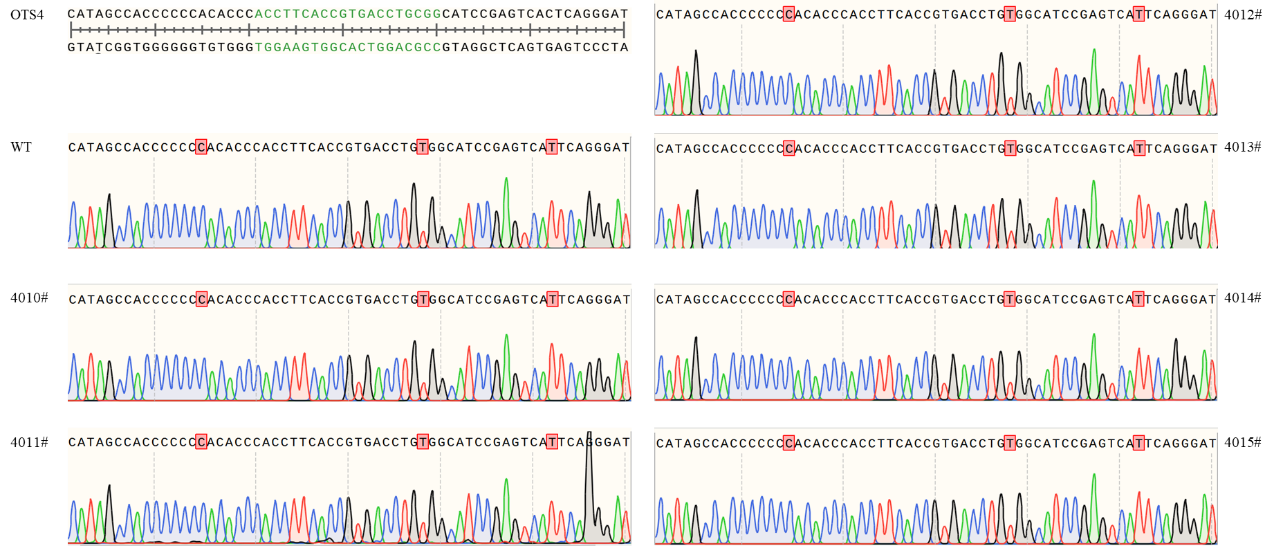


**Supplementary Figure S4. Sequencing results of OTS4 (NC_010446.5:7949354-7949376)**


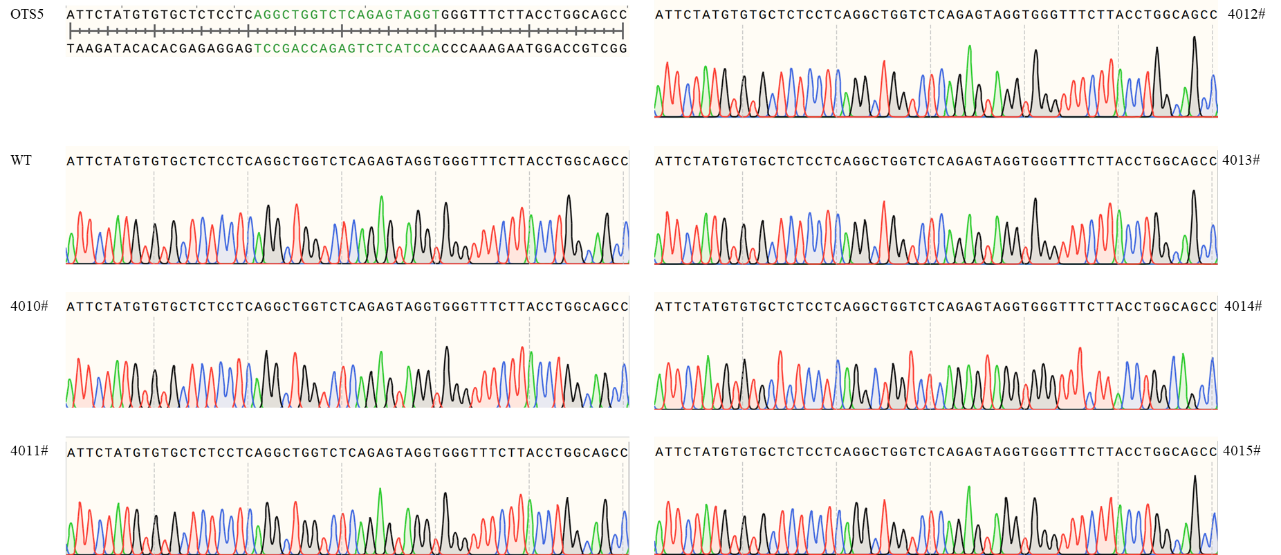


**Supplementar**y **Figure S5. Sequencing results of OTS5 (NC_010446.5:87054022-87054044)**


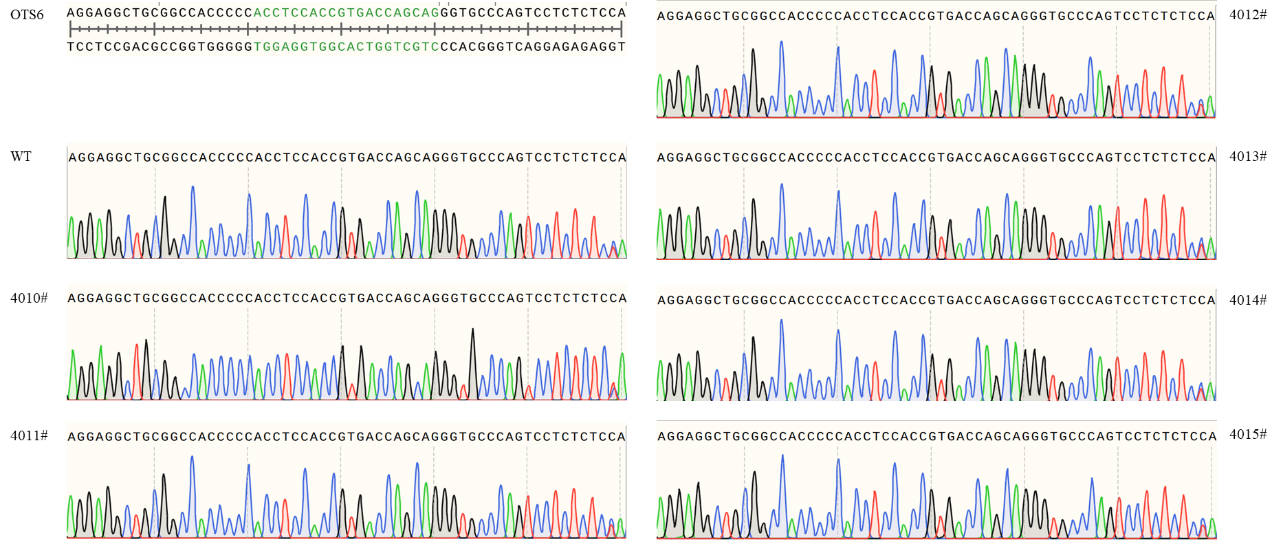


**Supplementar**y **Figure S6. Sequencing results of OTS6 (NC_010455.5:74539055-74539077)**


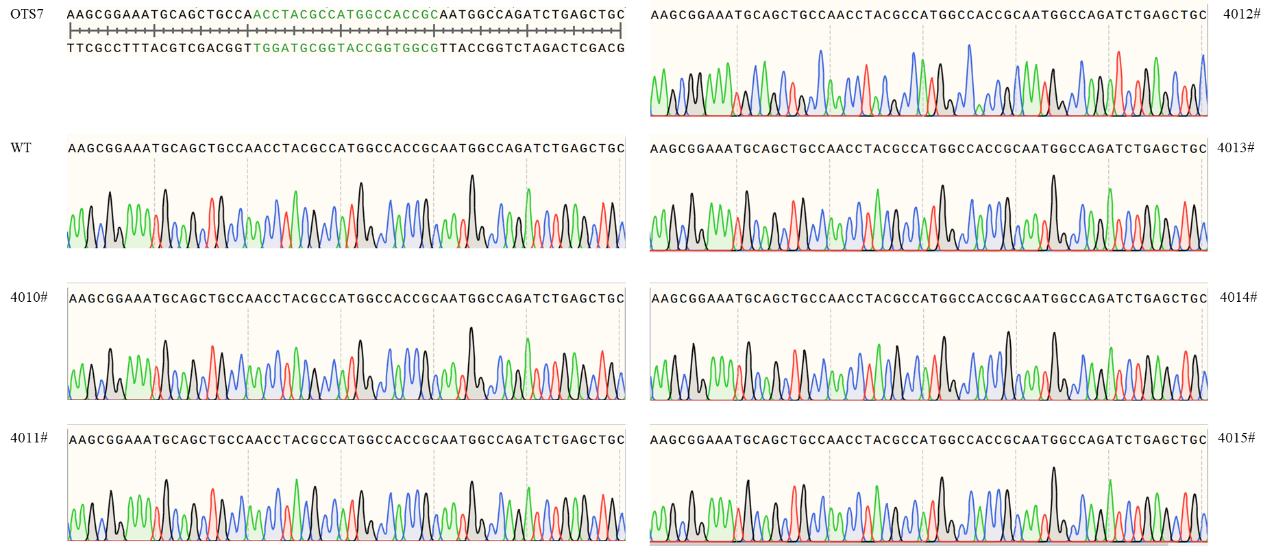


**Supplementar**y **Figure S7. Sequencing results of OTS7 (NC_010447.5:87463056-87463078)**


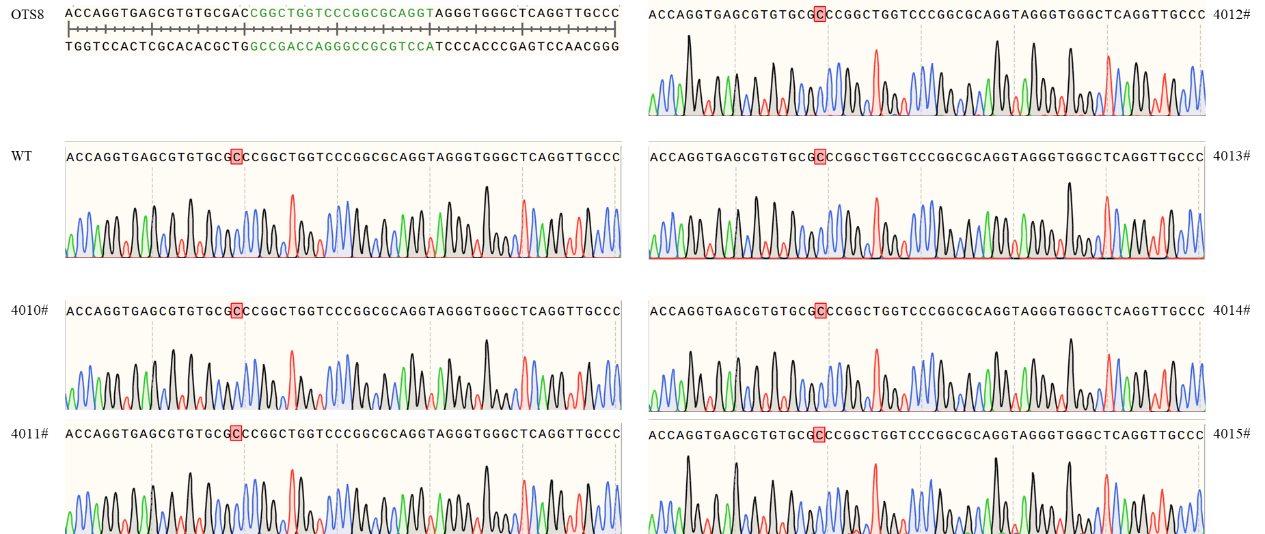


**Supplementar**y **Figure S8. Sequencing results of OTS8 (NC_010443.5:148035114-148035136)**


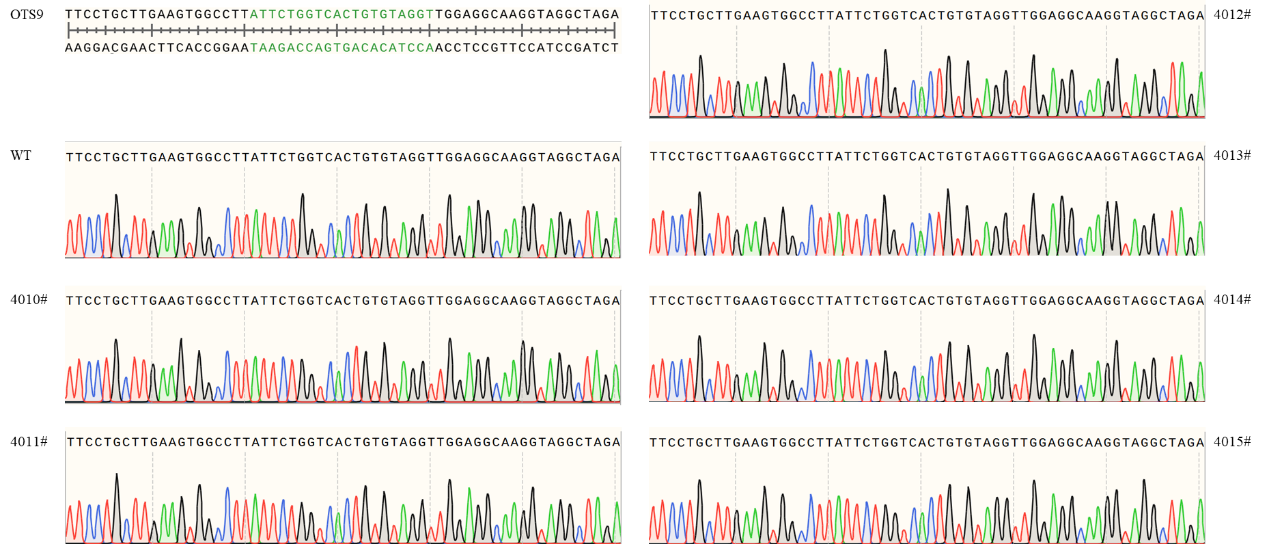


**Supplementar**y **Figure S9. Sequencing results of OTS9 (NC_010445.4:80076722-80076744)**


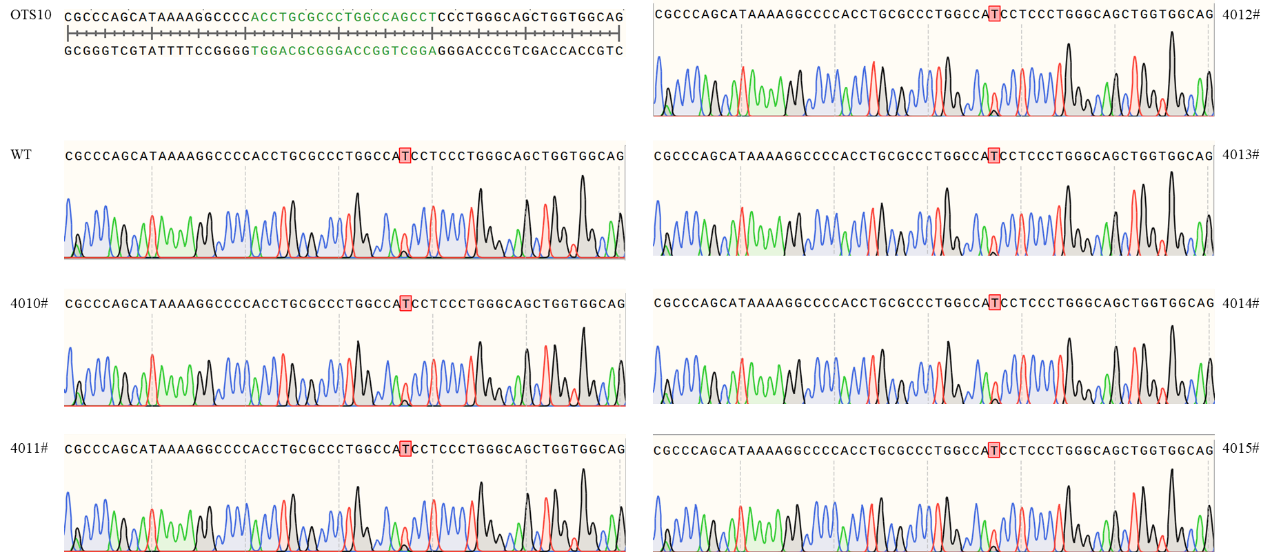


**Supplementar**y **Figure S10. Sequencing results of OTS10 (NC_010448.4:1557525-1557547)**


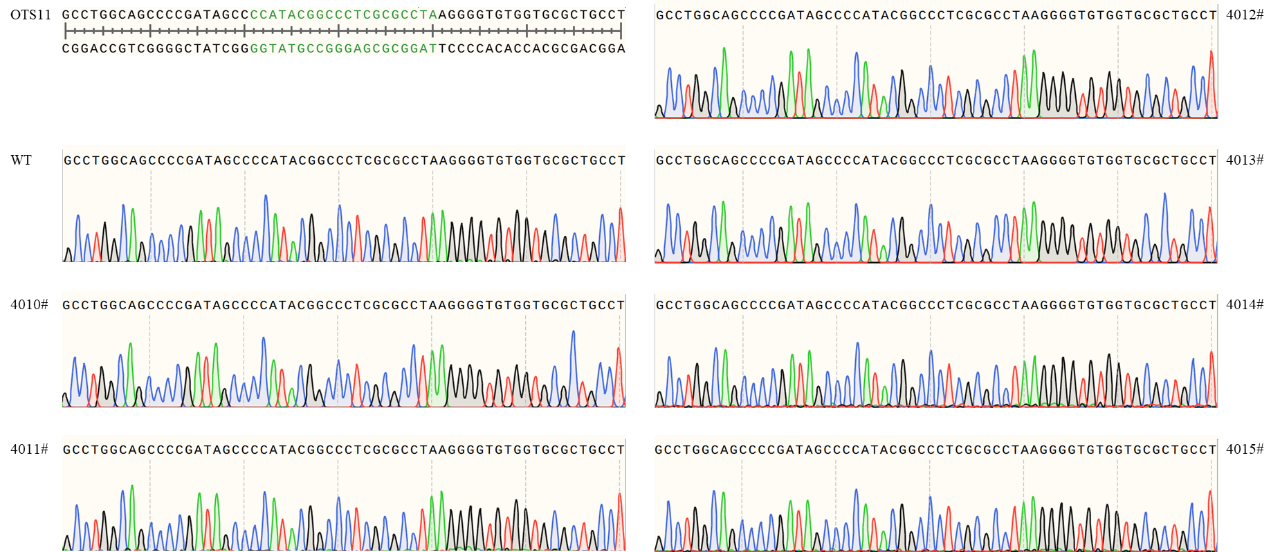


**Supplementary Figure S11. Sequencing results of OTS11 (NC_010450.4:84812512-84812534)**


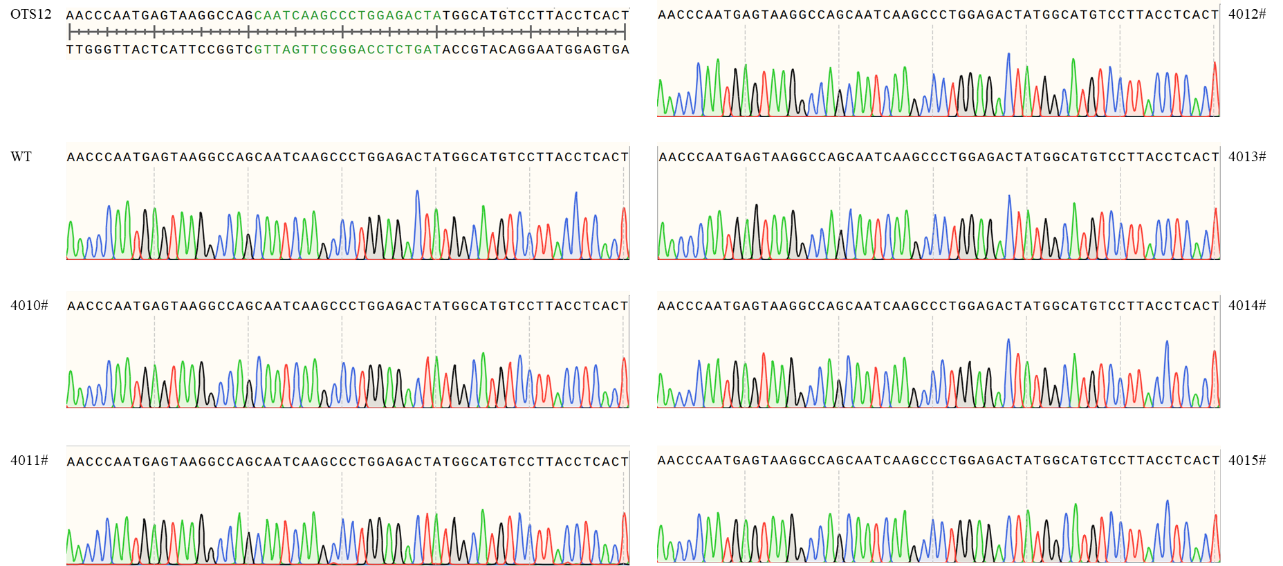


**Supplementary** **Figure S12. Sequencing results of OTS12 (NC_010443.5:205797259-205797281)**


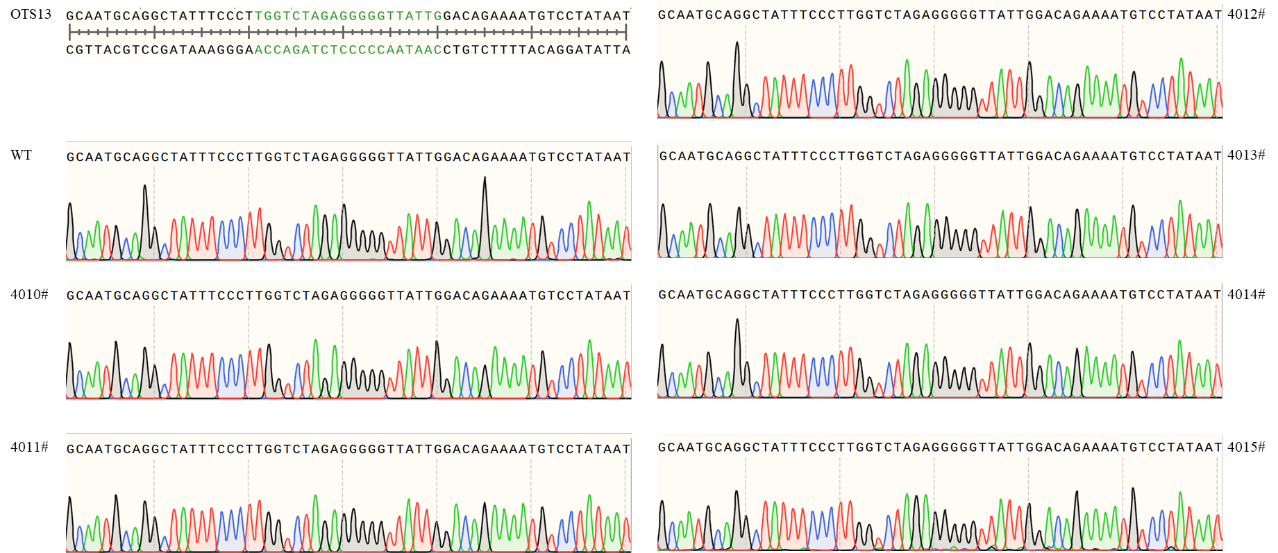


**Supplementary** **Figure S13. Sequencing results of OTS13 (NC_010443.5:157621551-157621573)**


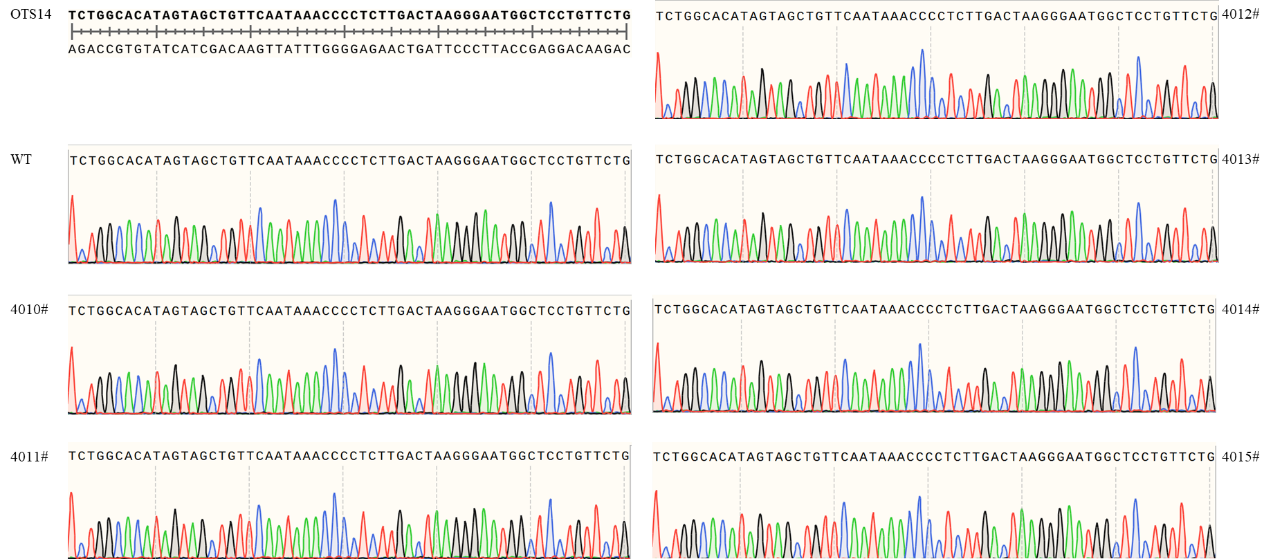


**Supplementary** **Figure S14. Sequencing results of OTS14 (NC_010455.5:70618532-70618554)**


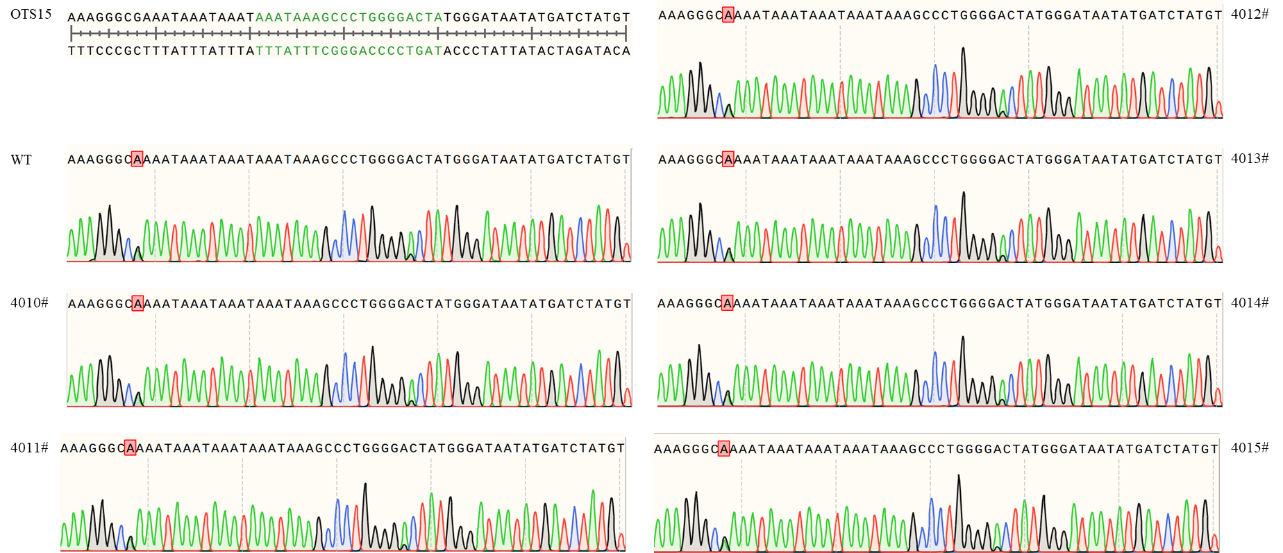


**Supplementary** **Figure S15. Sequencing results of OTS15 (NC_010449.5:44693010-44693032)**


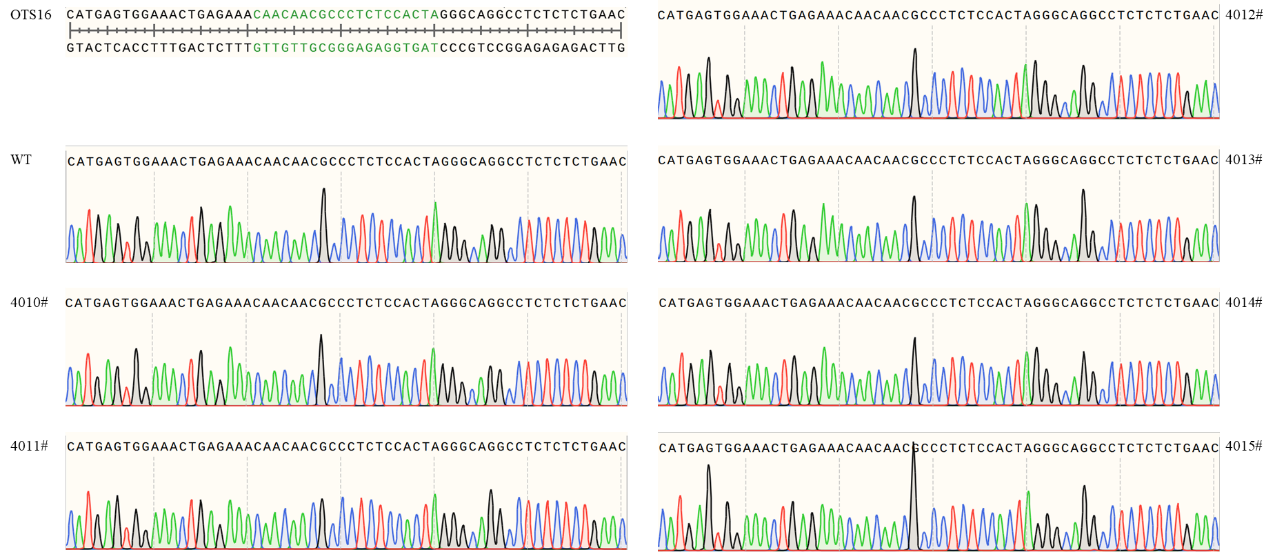


**Supplementary** **Figure S16. Sequencing results of OTS16 (NC_010457.5:87268870-87268892)**


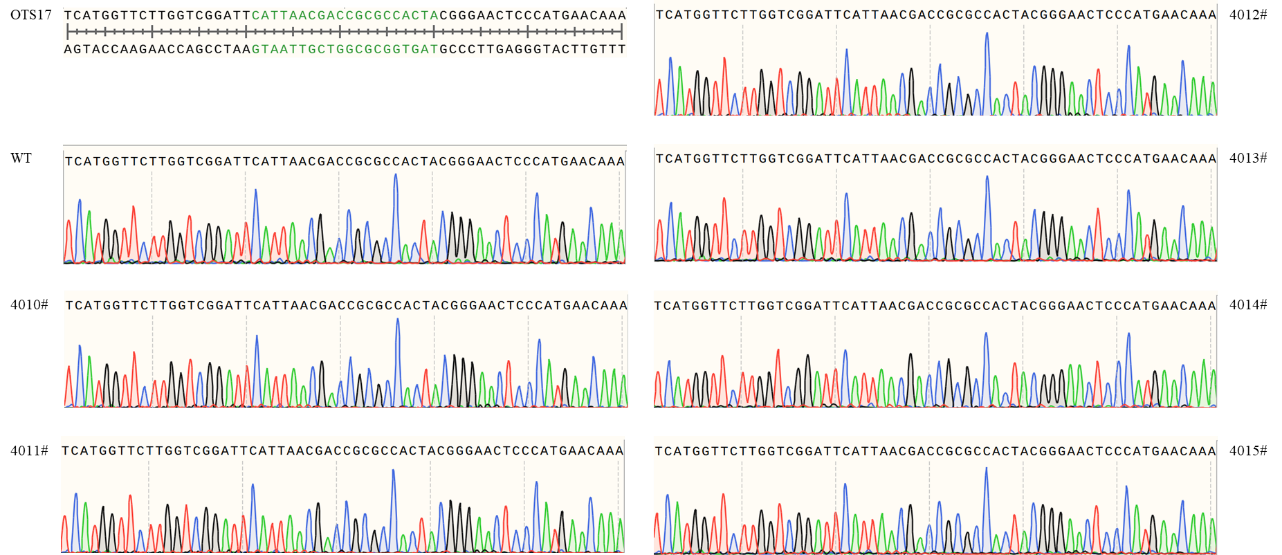


**Supplementary** **Figure S17. Sequencing results of OTS17 (NC_010445.4:118917258-118917280)**


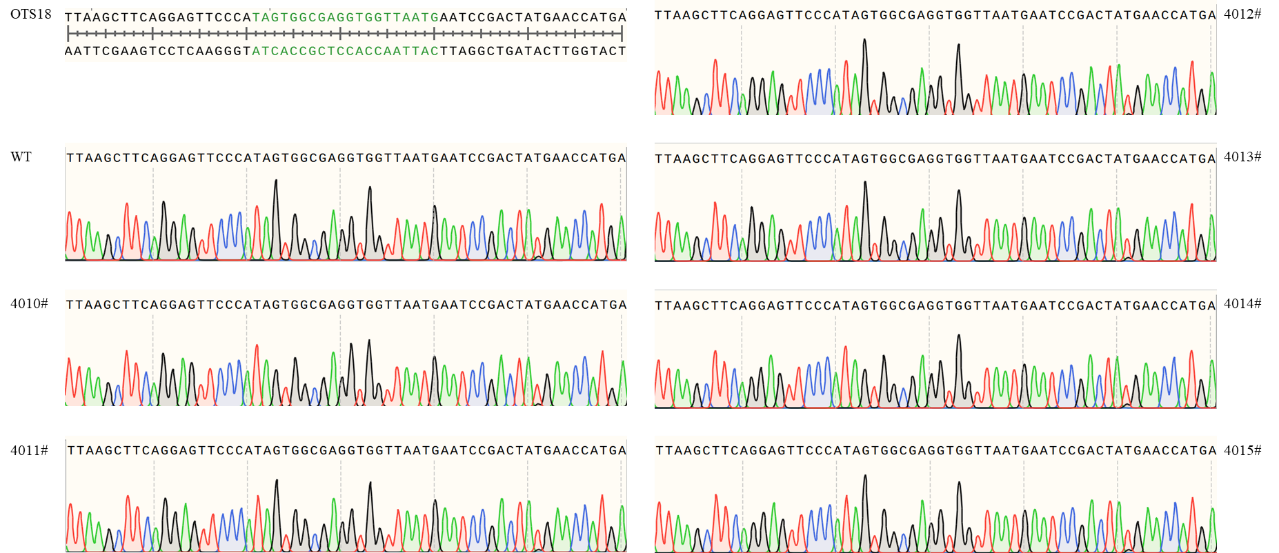


**Supplementary** **Figure S18. Sequencing results of OTS18 (NC_010449.5:38583621-38583643)**


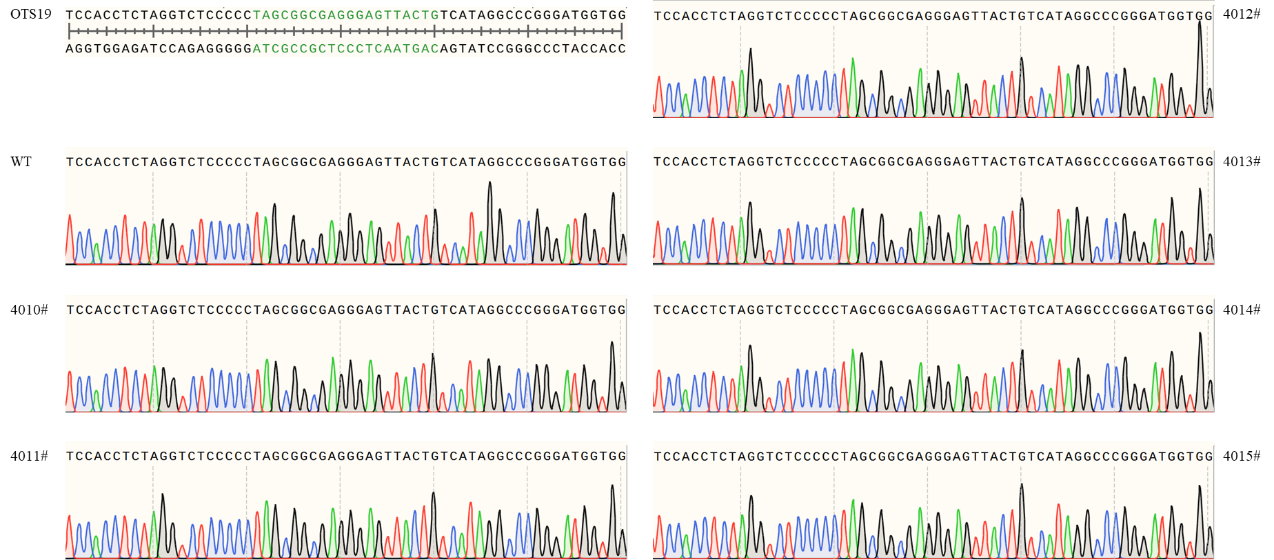


**Supplementary Figure S19. Sequencing results of OTS19 (NC_010449.5:116900169-116900191)**
